# Supplementary figures and images for: Risk Factors Associated with Hepatitis C Subtypes and the Evolutionary History of Subtype 1a in Mexico
Source: Viruses. 2024 Aug 6;16(8):1259. doi: 10.3390/v16081259 (PMC11359553; doi:10.3390/v16081259)

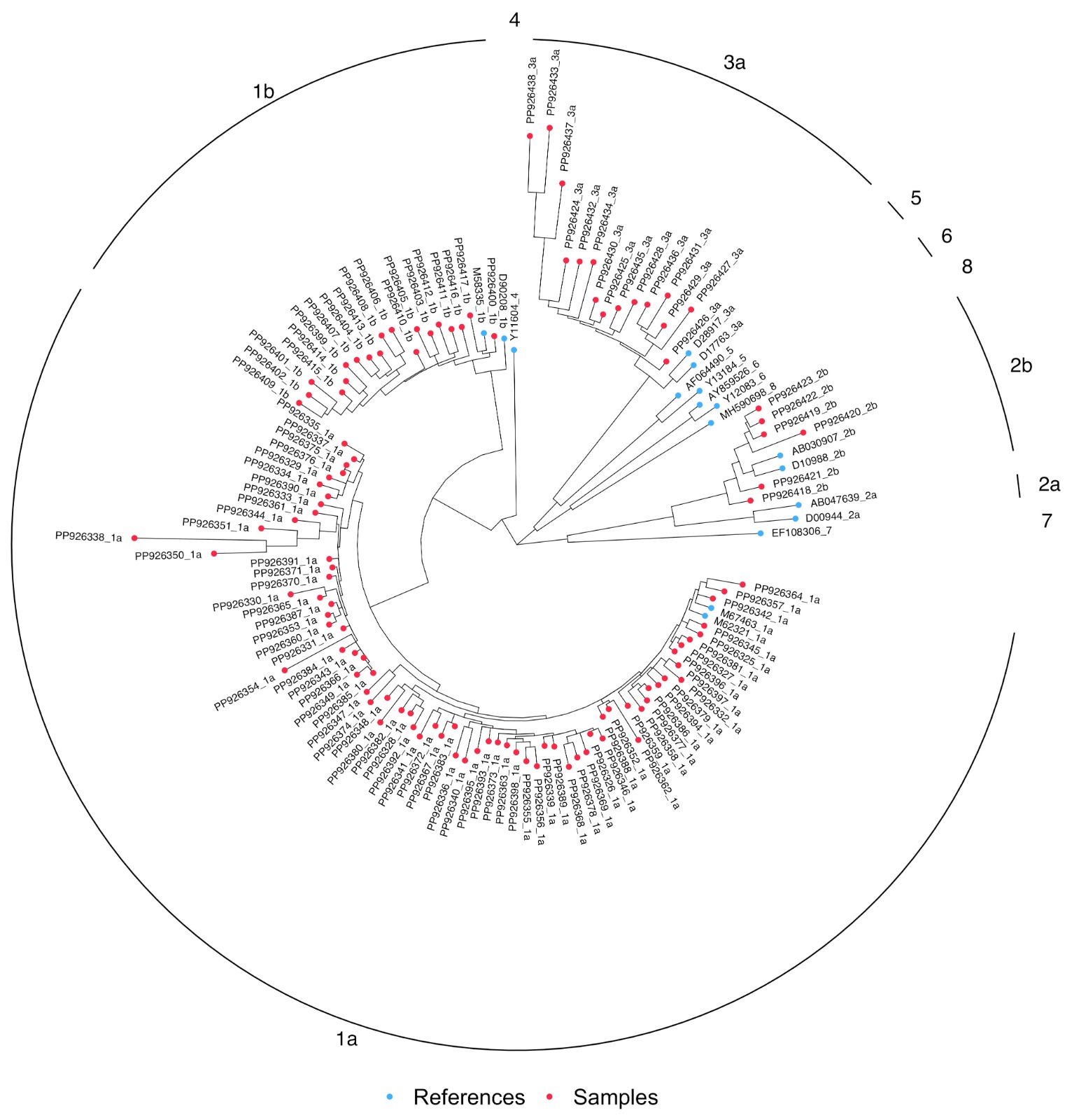

Supplement: Supplementary file 1 [file viruses-16-01259-s001.zip › S1_Figure_Phylogenetic Tree.jpg]
